# New Viruses from the Ectoparasite Mite *Varroa destructor* Infesting *Apis mellifera* and *Apis cerana*

**DOI:** 10.3390/v11020094

**Published:** 2019-01-24

**Authors:** Sofia Levin, Noa Sela, Tal Erez, David Nestel, Jeffery Pettis, Peter Neumann, Nor Chejanovsky

**Affiliations:** 1 Department of Entomology, The Robert H. Smith Faculty of Agriculture, Food and Environment, The Hebrew University of Jerusalem, Rehovot 76100, Israel; whirlingsofia@gmail.com; 2 Department of Entomology Institute of Plant Protection, Agricultural Research Organization, Rishon LeZion 7528809, Israel; tal.erez2@mail.huji.ac.il (T.E.); nestel@volcani.agri.gov.il (D.N.); 3 Department of Plant Pathology and Weed Research, Institute of Plant Protection, Agricultural Research Organization, Rishon LeZion 7528809, Israel; noa@volcani.agri.gov.il; 4 Institute of Bee Health, Vetsuisse Faculty, University of Bern, 3003 Bern, Switzerland; Pettis.jeff@gmail.com (J.P.); peter.neumann@vetsuisse.unibe.ch (P.N.)

**Keywords:** honeybee subspecies, *Varroa destructor*, metagenomics analysis, viruses

## Abstract

*Varroa destructor* is an ectoparasitic mite of Asian or Eastern honeybees *Apis cerana* *(A. cerana)* which has become a serious threat to European subspecies of Western honeybees *Apis mellifera* (*A. mellifera*) within the last century. *V.* *destructor* and its vectored honeybee viruses became serious threats for colony survival. This is a short period for pathogen- and host-populations to adapt. To look for possible variation in the composition of viral populations we performed RNA metagenomic analysis of the Western honeybee subspecies *A. m. ligustica*, *A. m.* *syriaca*, *A. m. intermissa*, and *A. cerana* and their respective *V. destructor* mites. The analysis revealed two novel viruses: Varroa orthomyxovirus-1 (VOV-1) in *A. mellifera* and *V. destructor* and a Hubei like-virga virus-14 homolog in *V. destructor*. VOV-1 was more prevalent in *V. destructor* than in *A. mellifera* and we found evidence for viral replication in both hosts. Interestingly, we found differences in viral loads of *A. cerana* and their *V. destructor*, *A. m. intermissa*, and its *V. destructor* showed partial similarity, while *A. m.* *ligustica* and *A. m.* *syriaca* and their varroa where very similar. Deformed wing virus exhibited 82.20%, 99.20%, 97.90%, and 0.76% of total viral reads in *A. m. ligustica*, *A. m. syriaca*, *A. m. intermissa*, and *A. cerana,* respectively. This is the first report of a complete segmented-single-stranded negative-sense RNA virus genome in honeybees and *V. destructor* mites.

## 1. Introduction

The mite *Varroa destructor* is an obligatory ectoparasite of the Eastern honeybee *Apis cerana* [1]. *V. destructor* mites spend most of their life cycle inside the colony, reproducing on the honeybee brood and feeding on the pupa. Mites transfer from one host pupa to another on nurse bees that take care of pupa [1]. During the feeding on its host this mite may transfer populations of microorganisms that it bears, and viruses in particular, as well as acquire those that belong to the host [2,3,4,5]. *V. destructor* made a host shift to the Western honeybee *A. mellifera* at the beginning of the 20^th^ century and spread to Europe, USA, New Zealand, Africa, and the Middle East from southern and southeastern Asia during the last century [6].

Viruses are important pathogens of the honeybee. Different studies reported that Acute bee paralysis virus (ABPV), Deformed wing virus (DWV), Israel acute paralysis virus (IAPV), Kashmir bee virus (KBV), Sacbrood virus (SBV), Slow bee paralysis virus (SBPV), Chronic bee paralysis virus (CBPV), Lake Sinai virus (LSV), Apis mellifera filamentous virus (AmFV), and Black queen cell virus (BQCV) are pathogenic to honeybees [4,7,8,9,10,11].

Some of the above viruses DWV, SBPV, ABPV, IAPV, and KBV—are transmitted by *V. destructor*, which has become a growing threat to the existence of *A. mellifera* [2,3,12,13,14,15]. *V. destructor* and viral pathogens play a key role in the loss of *A. mellifera* colonies, especially because both the mite and the viruses produce a double effect in colony infestation: *V. destructor* being tightly linked to honeybee life cycle causes a considerable impact on the brood by feeding on it and transmitting viruses [4,16,17,18]. Viruses being vectored by mites and thus directly introduced into the pupa’s body during feeding might reach noticeably higher titers in developing honeybees [17,19,20]. Together they cause a higher impact on honeybee colony health and even induce its collapse [12,21,22].

From an evolutionary point of view, the shift of *V. destructor* from *A. cerana* to *A. mellifera* is a relatively short period of time for the populations of the pathogen and the host to adapt and thus, *A. mellifera* is a new host for *V. destructor*. This is valid for its viral content as well. To look for possible variations in virus composition we investigated the viral populations in samples of three *A. mellifera* subspecies from the Middle East and North Africa (MENA) regions and their parasitic *V. destructor* mites as well as from *A. cerana* and their *V. destructor* mites. For this purpose we used next-generation sequencing (NGS) and performed metagenomics analysis of viral populations from samples of *A. m. ligustica* colonies from Israel [23] and their *V. destructor* mites, from Western honeybee subspecies from the MENA region—*A. m. intermissa* and *A. m. syriaca* [24]—and their *V. destructor* mites, and *A. cerana* and their *V. destructor* mites from Thailand. In this study, the viral composition and loads from samples of each bee species and its mite were determined, viral loads and viruses of interest were validated and characterized using molecular biology tools. This analysis indicated differences in viral composition and load in the samples of the above honeybee subspecies and their parasitizing varroa mites. We found that in these samples *A. cerana* and their parasite *V. destructor* differed considerably in their viral load, *A. m. intermissa* and its varroa mites showed partial similarity in their viral load, while *A. m. ligustica* and *A. m. syriaca* and their corresponding varroa mites’ viral loads were more similar. Moreover, besides identifying known and recently discovered viruses, we found two novel viruses—an Orthomyxovirus common to *A. mellifera* and *V. destructor*—that we named Varroa orthomyxovirus-1 (VOV-1) and a homolog of the Hubei like-Virga virus 14 that we named VDV-4. VOV-1 showed 23–58% homology to the Orhomyxoviruses Thogoto and Dhori (THOV and DHOV), which bear negative-sense single stranded RNA genomes of six segments [25,26,27,28]. Replication of single-stranded sense RNA viruses requires the synthesis of the complementary positive-sense RNA, which can be detected by using strand-specific RT-PCR [29]. Using this approach we were able to demonstrate replication of VOV-1. This is the first report of the presence of a segmented negative-sense ssRNA virus in honeybees and varroa mites.

## 2. Materials and Methods

### 2.1. Sample Collection

The experimental colonies of *A. m. ligustica* (worker bees *N* = 48, from hives 1, 3, 5, and 23, (4 bees per hive), 14 (6 bees), 81 (9 bees), and 401 (1 bee); mites *N* = 606, from the same hives (85 of them from emerging bees and the rest from free falling mites), collected between October to February 2016, were described before [23]; *A. m. syriaca* (workers *N* = 15, from a subcollection of 500 workers from colonies from several apiaries; mites = 20, from tens of mites that were collected in 2013) and *A. m. intermissa* (workers *N* = 15, from a subcollection of 500 workers; *V. destructor* mites *N* = 27, from tens of mites that were collected in 2013) were described previously [24]. *A. cerana* drones (*N* = 6) and *V. destructor* mites (*N* = 20) were sampled during spring 2016 (December) in Phrae, Thailand from two colonies 3c and 4c untreated against mites (3 drones and 20 mites from each colony, respectively). The honeybees and corresponding mites were transported in RNA *later*™ and stored immediately at −80 °C until RNA extraction.

### 2.2. Samples Preparation

RNA extraction from all *A. mellifera* and mite samples was carried out using TRI Reagent^®^ (Sigma-Aldrich, Israel) according to the manufacturer’s instructions as published before [24,30]. RNA from *A. cerana* drones and the corresponding *V. destructor* mites was individually extracted using a GenJet RNA purification Kit (Thermo Scientific, Burlington, Canada) according to the manufacturer’s instructions.

### 2.3. Transcriptome and Virome Analysis

Construction and paired-end sequencing of the libraries from *A. cerana* and its corresponding *V. destructor* mites-RNA samples was performed at the Technion Genome Center on a HiSEq 2000 platform (Illumina, Haifa, Israel). Paired-end reads were assembled de novo using Trinity [31]. The obtained contigs were translated and aligned to the GenBank nonredundant (NR) database by Blastx [32]. Next-generation sequencing (NGS) of the RNA from *A. m. ligustica*, *A. m. intermissa*, and *A. m. syriaca* honeybees and corresponding *V. destructor* mites was described previously [23,24]. Metagenomic analysis of *A. m. ligustica*, *A. m. intermissa*, and *A. m. syriaca* subspecies and *A. cerana* bees, and of *V. destructor* mites samples were carried out as described previously and in Section 2.8 [30].

### 2.4. RT-PCR

cDNA was prepared using RevertAid Reverse Transcriptase (Thermo Scientific) with oligo-dT and random primers according to the manufacturer’s instructions. One-hundred nanogram and 2000 ng RNA templates were used from *V. destructor* and honeybee samples, respectively. RT-conditions: incubation of RNA and primers at 65 °C for 5 min., followed by addition of buffer containing 50 mM Tris-HCl (pH 8.3), 75 mM KCl, 2 mM MgCl_2_, 5 mM DTT, 4 units of RNase inhibitor Ribolock^®^ (Thermo Scientific), and the RT enzyme (200 units) in a 25 μL volume, and further incubation at 55 °C for 30 min. The reaction was terminated by heating at 85 °C for 5 min. PCR-validations were performed with GoTaq^®^ (Promega Corporation, Madison, WI, USA) using 1 μL cDNA template and 0.2 μM of each forward and reverse primer in a 20 μL reaction with the following conditions; 95 °C for 4 min, 32 cycles at 94 °C for 30 s, then 56 °C (VOV-1 segments 1,2,4,5) or 57 °C (VOV-1 segments 3,6) for 50 s, 72 °C for 2 min. (VOV-1 segments 1,2,4,5) or 1 min. (VOV-1 segments 3,6), and a final extension step of 72 °C for 10 min. For VDV-4 the PCR conditions were identical to those used in segments 1, 2, 4, 5 of VOV-1 with the corresponding specific primers. Specific primers used for validations are described in the Tables S1 and S2 in the Supplementary material.

### 2.5. VOV-1 Prevalence

VOV-1 prevalence was determined by using RT-PCR to detect the presence of the segment 6 of the virus genome in samples of *V. destructor* mites and honeybees in apiaries located at the North (Haifa, Kibbutz Lehavot HaBashan, Kibbutz Dan), the Center (ARO, Nitzanei Oz, Herut, Kfar Ruth) and the South (Kibbutz Yad Mordechai) of Israel.

### 2.6. qRT-PCR

Viral genome copy number was quantified on a PikoReal 96 machine (Thermo Scientific) using a standard protocol (95 °C 2 min; 40 cycles of 95 °C 10 s, 60 °C 20 s, 72 °C 20 s). Each quantitative PCR analysis was performed in triplicate. Nontemplate controls (water) were included in triplicates in each assay. The KAPA SYBR FAST qPCR Master Mix (2×) Universal (Kapa Bio-systems) was used, in a 10 μL final volume. For each analysis 2 μL of the diluted cDNA was used (dilution factor of 4) and specific primers VOV-1-qRT-F1 and VOV-1-qRT-R1 at a concentration of 0.25 μM each (Table S1). The specificity of the amplicons synthesized during the PCR run was ascertained by performing a dissociation curve protocol from 60 °C to 95 °C. Specific primers used for quantification are provided in Table S1 in Supplementary material.

### 2.7. Replication Assay

Testing for viral replication (presence of the positive strand-sense RNA) was performed by synthetizing the negative-strand cDNA of fragment 6 from the RNA samples using the tagged primer VOV6-46F-TAG, as we described before to analyze replication of BRV-1 [30]. Subsequently the residual VOV6-46F-TAG primer was inactivated by adding to the mixture exonuclease-I and incubating it for another 15 min. at 37 °C (method described in de Miranda et al, 2013 [33]). Finally the exonuclease I was inactivated by heating the mixture at 80 °C for 15 min. Subsequently, PCR was performed with primers VOV6-870R and TAG (Supplementary material 1 Table S1). cDNA produced without any primer was used as control in the same reactions followed by PCR with the same primers as above. PCR was performed at 95 °C for 4 min., 30 cycles at 94 °C for 30 s, then 58 °C for 50 s, 72 °C for 1 min. and a final extension step of 72 °C for 10 min. The identity of the amplified fragment was confirmed by Sanger sequencing (performed at the Biological Services Unit of the Weizmann Institute of Science, Israel).

### 2.8. Bioinformatic Identification of Contigs

Each RNAseq library was de novo-assembled using Trinity assembler version 2.2.0 [31]. The assembled contigs were then searched with BLASTX [33] against the NCBI nonredundant protein database (NR) [34]. After the assembled viruses were identified in each library, each library’s raw data reads were mapped using bowtie2 [35] to evaluate the virus quantity in the transcriptome. We reanalyzed the data obtained before in *A. m. ligustica* [23] and realized that contigs of a new Orthomyxovirus were present. Then we analyzed data that we downloaded from recently published transcriptomes of viruses of *A. m. intermissa* and *A. m. syriaca* and their varroa from the MENA region [24] and were able to assemble the complete genome of the virus (Table 1 and Table 2).

### 2.9. Molecular Phylogenetic Analysis

Phylogenetic analysis was done using MEGA 6 [34]. Alignment of the proteins was done using MAFFT [36], and then Maximum likelihood Phyml 3.0 was used for creating the tree [37] with a 100 bootstrap.

## 3. Results

### 3.1. Metagenomic Analysis of Viruses in A. mellifera, A. cerana and Their V. destructor Mites

In the analysis we included libraries from *A. m. ligustica*, *A. m. syriaca*, *A. m. intermissa*, *A. cerana*, and their *V. destructor* counterparts (see Materials and Methods). This revealed variation in composition of the viral loads of *A. m. ligustica*, *A. m. syriaca*, *A. m. intermissa*, *A. cerana*, and their *V. destructor* mite counterparts (libraries IB1, SB2, AB3, BCER, IV4, SV5, AV6, and VCER, respectively). Mapping of the libraries’ reads to viral contigs of honeybees and *V. destructor* mites resulted in

1. identification of the most common honeybee viruses: Acute bee paralysis virus (ABPV), Israeli acute paralysis virus (IAPV) in IB1, IV4, and SB2; Apis mellifera filamentous virus (AmFV) in AB3; Bee Macula-like virus/Varroa Macula-like virus (BeeMLV/VdMLV) and Black queen cell virus (BQCV) in all the libraries except for BCER and VCER; Deformed wing virus (DWV) in all the libraries; Lake Sinai virus (LSV) in IB1; and Sacbrood virus (SBV) in IB1, IV4, SV5, and AV6 (details are provided in Materials and Methods and in Table 3).
2. identification of recently described viruses Apis rhabdovirus-1/Bee rhabdovirus-1(ARV-1/BRV-1) [30], Varroa destructor virus-2 (VDV-2), and Varroa destructor virus-3 (VDV-3) [23].
3. discovery of two new viruses that we designed Varroa orthomyxovirus-1 (VOV-1), an orthomyxovirus with low homology to other viruses from the Orthomyxoviridae family, and Varroa destructor virus-4 VDV-4 (Table 3 and see below).

Interestingly, ARV-1/BRV-1 and VOV-1 showed differences in their distribution across bee and *V. destructor* libraries; while ARV-1/BRV-1 was present in all *V. destructor* libraries and in two of the honeybee libraries, VOV-1 was limited to *A. m. ligustica* and *A. m. syriaca* and their corresponding varroa (Table 3).

**Figure 1:**
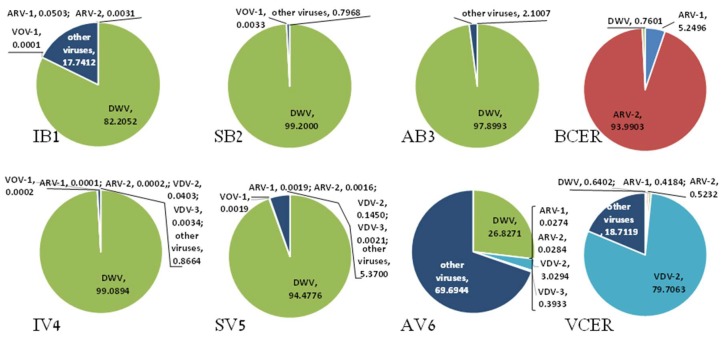
Presence of viruses in honeybee and *V. destructor* mite by library. Percentage of total viral reads* mapping to viral contigs of the honeybees and *V. destructor* mites libraries. *Including: DWV and genetic variants, ARV-1, ARV-2, VOV-1, VDV-2, VDV-3, and other viruses (11 more, see Table 1), cutoff at 0.0001%.

The presence of viruses varied across *A. mellifera* and *A. cerana* bees and their corresponding *V. destructor* libraries. From the *A. mellifera* libraries analyzed, IB1 and IV4 (*A. m. ligustica*) showed a similar percentage of viral reads of DWV, as did SB2 and SV5 (*A. m. syriaca*) and *A. m. intermissa*, and their *V. destructor* libraries—AB3 and AV6—showed large differences (Figure 1). Also, *A. cerana* libraries—BCER and VCER—were distinct (Figure 1 and see below). For instance, AV6 showed a smaller percentage of DWV reads compared to AB3 and to the other *A. mellifera*—IB1 and SB2—and their corresponding *V. destructor* libraries—IV4 and SV5, respectively (Figure 1). The cDNA libraries of *A. cerana* (BCER) and its varroa (VCER) displayed extremely low percentage of viral reads for DWV (0.7601% and 0.6402%, accordingly). As can be seen, our samples BCER and VCER differed in their load of other viruses as well; while the BCER main viral component was ARV-2 [38], the main virus present in VCER was VDV-2 (Figure 1). In addition, VCER displayed two viruses present in varroa parasites of *A. cerana* only with 38% homology to Hubei picorna-like virus 29 [39] and 36% homology to Hubei virga-like virus 14 [40] (Table 3 and see below).

### 3.2. Varroa Orthomyxovirus-1 and the Hubei Virga-like 14 Homolog Virus

We reanalyzed the data obtained before in *A. m. ligustica* [23] and realized that contigs of a new Orthomyxovirus were present in the data. To complete the picture we downloaded and analyzed data from the transcriptome of viruses of *A. m. intermissa* and *A. m. syriaca* and their varroa mites from the Middle East and North African (MENA) honeybees and varroa mites that were published recently but did not focus on finding new viruses [24]. This additional sequence data facilitated complete genome assembly of this new virus. According to BLASTX analysis we identified VOV-1 contigs in two cDNA libraries of bees (IB1 and SB2) and two cDNA libraries of their corresponding varroa mites (IV4 and SV5). VOV-1 showed 23–58% homology to the Orthomyxoviruses Thogoto and Dhori (THOV and DHOV), which bear negative-sense single stranded RNA genomes of six segments [25,26,27,28]. Contigs of 2198, 1899, and 358 nucleotides in length from the SV5 library showed homology of 58%, 29%, and 46% to polymerase subunits PB2, PB1, and PA encoded in segments 1, 2, and 3 of the DHOV genome, respectively (Table 4). A contig of 232 nucleotides length from the IV4 library showed homology of 41% to the glycoprotein subunit (GP) encoded in segment 4 of THOV; a contig of 1442 nucleotides length from IB1 library showed homology of 39% to the nucleoprotein subunit (NP) encoded in segment 5 of DHOV; and a contig of 983 nucleotides from the SB2 library showed homology of 23% to matrix protein (M) encoded in segment 6 of THOV (Table 4).

**Figure 2:**
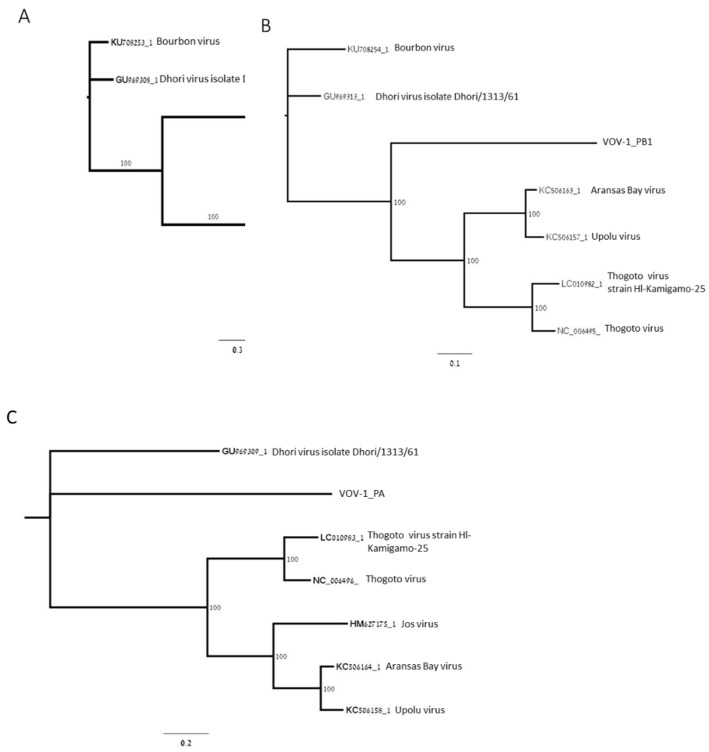
Maximum-likelihood phylogenetic tree of VOV-1 contigs for segments 1, 2, and 3. The trees were constructed based on the ORF of VOV-1 polymerase subunits PB2 (**A**), PB1 (**B**), and PA (**C**). GeneBank accession numbers provided.

Phylogenetic analysis of open reading frames (ORFs) coding for polymerase subunits PB2, PB1, and PA showed that the polymerase was closely related to negative-sense ssRNA viruses belonging to the Orthomyxoviridae viral family: Thogoto virus (THOV); Aransas Bay virus (ABV); Upolu virus (UPOV) (Figure 2A–C); and Jos virus (JOSV) (Figure 2A,C). PB2 is phylogenetically closer to THOV, ABV, UPOV, and JOSV, and more distant from DHOV and Bourbon virus (BRBV) as well as PB1, except for JOSV, and PA, except for BRBV (Figure 2A–C).

**Figure 3:**
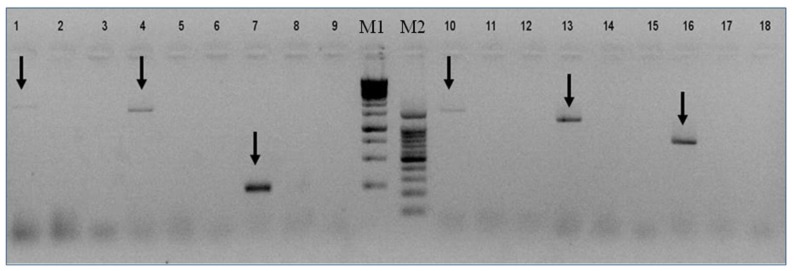
Presence of VOV-1 in virome of *V. destructor* mites and its absence in honeybees. *V. destructor* mite virome: lanes 1, 4, 7, 10, 13, and 16; Honeybee virome: lanes 2, 5, 8, 11, 14, and 17; NTC (nontemplate control): lanes 3, 6, 9, 12, 15, and 18; M1 and M2: GeneRuler Marker^TM^ 1 kb, 100 bp DNA ladders, respectively. Arrows: VOV-1 segments (1–6).

Based on the above contigs’ sequences we designed specific primers to validate the presence of each segment of VOV-1 in the viromes of Israeli *A. mellifera ligustica* and their counterpart *V. destructor* parasites (Figure 3 and see Materials and Methods). All six segments were identified in the *V. destructor* virome (Figure 3, lanes 1, 4, 7, 10, 13, and 16) but they were absent in the virome of honeybees (Figure 3, lanes 2, 5, 8, 11, 14, and 17).

Furthermore, we tested the presence of segment 6 of the viral genome to estimate the prevalence of VOV-1 by RT-PCR in individual mites and honeybees. We detected VOV-1 in 35.56% of *V. destructor* mites from Israeli colonies located in ARO, Beit Dagan (16 of 45), and in none of 32 individual honeybees sampled from the same colonies. We also analyzed its prevalence in colonies located in the Center, North, and South of Israel by testing pools of the honeybees and *V. destructor* mites with the same PCR method. The virus was identified in 78.57% of *V. destructor* pools (11 of 14 pools) and only in 8.33% of honeybee pools (5 of 60 pools).

The number of genomic copies of VOV-1 estimated by qRT-PCR was similar in individual mites collected from honeybee colonies in ARO, Beit Dagan, in pools of mites, and in honeybee pools sampled from colonies located in the Center, North, and South of Israel: 5.11×10^2^–1.22×10^6^, 2.33×10^3^–4.88×10^5^, and 4.91×10^2^–1.38×10^5^, respectively (Table 5).

**Figure 4:**
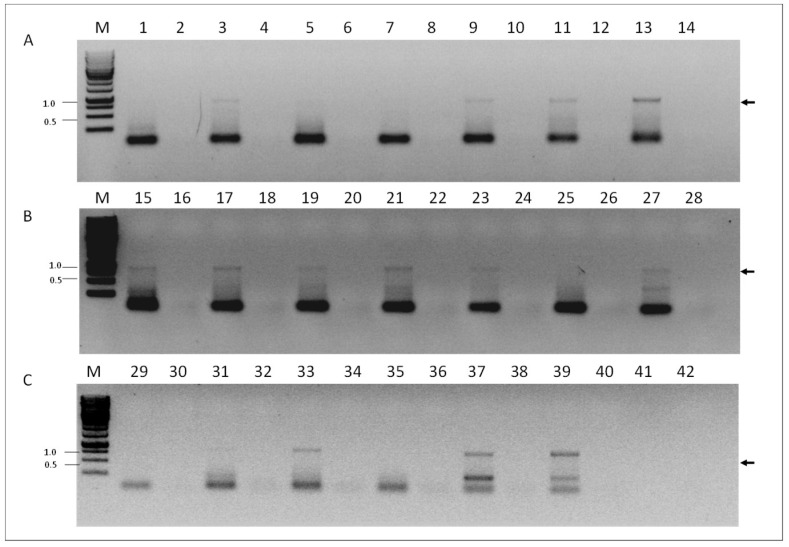
Detection of the VOV-1-segment 6 positive-sense RNA strand in *V. destructor* and *A. mellifera.* (**A**) *V. destructor* individuals, odd sample numbers and their respective controls, even sample numbers. (**B**) *V. destructor* (pools) odd samples, and their respective controls, even samples. (**C**) Lanes 29 and 30, *V. destructor* (pool) and its respective control; *A. mellifera* (pools), odd lanes 31–39 and respective controls, even lanes 32–40. PCR control reaction of the same individual RNA performed on cDNA produced without any primer (see the section “Materials and Methods”. PCR primers: VOV6-870R and TAG. M, GeneRuler Marker 1 kb DNA Ladder (Thermo Scientific Inc.); 41 and 42: nontemplate control; Arrow, VOV-1 amplicon that was confirmed by sequencing (see “Materials and Methods”).

To investigate if VOV-1 replicates in our samples, we screened for presence of the positive-sense RNA strand of the fragment 6 of the virus using RNA-strand sense-specific primer-tagged RT-PCR (see Materials and Methods). A predicted size fragment of ~840 nucleotides corresponding to the VOV-1 positive-sense-strand RNA between nucleotides 46 and 870 was found in tested samples from individual *V. destructor* mites (Figure 4, panel A, lanes 3, 9, 11, and 13), or from *V. destructor* mite and nurse honeybee pools (Figure 4, panels B and C, lanes 15, 17, 19, 21, 23, and 27 and 33, 37, and 39, respectively). No amplification was observed in some varroa individuals, varroa and nurse honeybee pools (Figure 4, panels A, B and C, lanes 1, 5, and 7 and 25, 29, 31, and 35, respectively). Control samples obtained when PCR was performed with cDNA prepared from the same RNA without the corresponding oligonucleotide primer in the RT reaction did not show any amplicon (Figure 4, panels A, B, and C, lanes 2, 4, 6, 8, 10, 12, 14, 16, 18, 20, 22, 24, 26, 28, 30, 32, 34, 36, and 38, respectively).

We confirmed by Sanger DNA sequencing that the above specific-primer-tagged amplicons were identical to the VOV-1 sequence comprising nucleotides 46 and 870 of segment 6 of the viral genome.

**Figure 5:**
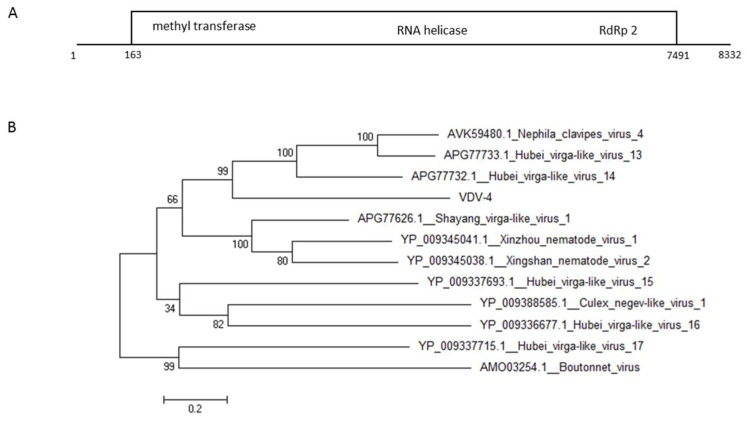
Genomic organization (**A**) and maximum-likelihood phylogenetic tree (**B**) of VDV-4. The tree was constructed based on the ORF of VDV-4. GeneBank accession numbers provided.

From the two undescribed viruses that we found in VCER we further investigated VDV-4. Phylogenetic analysis using the putative large ORF protein of the virus showed that it is 36% homologous to the hypothetical protein gene of spider viruses Hubei virga- like virus 14 and Hubei virga-like virus 13 as well as to the spider putative protein of the virus Nephila clavipes virus 4 (Figure 5).

**Figure 6:**
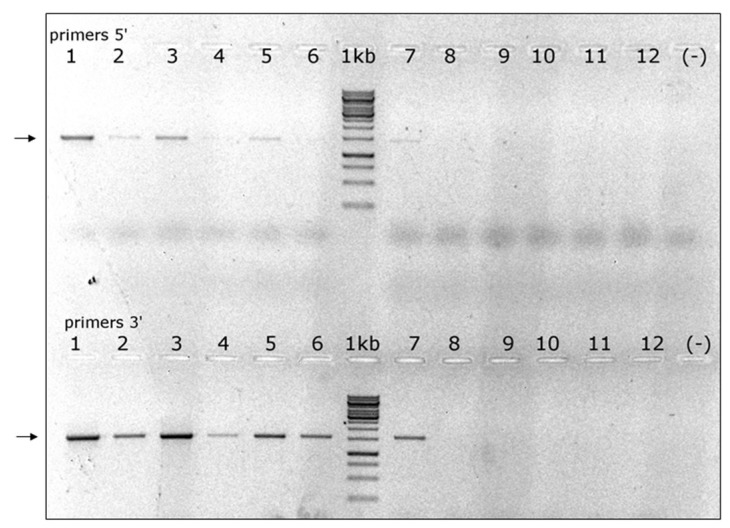
Presence of VDV-4 in *V. destructor* mites and *A. cerana* drones: lanes 1–3 and 4–6 pools of 5 *V. destructor* cDNAs each from colonies 3c and 4c, respectively; lanes 7–9 and 10–12 individual *A. cerana* drones, 3 per colony; lanes (−) nontemplate control; lanes 1 kb, GeneRuler Marker^TM^ 1 kb DNA ladder. Arrows: VDV-4, Upper panel VDV-4 genome 5’-end primers VDV4-1451F and VDV4-2889R, expected fragment 1438 bp; lower panel VDV-4 genome 3’-end primers VDV4-6242F and VDV4-7818R, expected fragment 1576 bp.

RT-PCR validation of its presence in a small sample of *A. cerana* drones and *V. destructor* individuals suggested that this virus was predominant in the latter (Figure 6). Therefore, we decided to name it VDV-4.

## 4. Discussion

We presumed that since *Apis mellifera* is a new host to *Varroa destructor* they might show differences both in the composition and distribution of their viral load. Our analysis of the data illustrates changes in viral composition and load among samples from *A. mellifera* subspecies and their *V. destructor*. Interestingly, a small-scale sample of *A. cerana* and its *V. destructor* showed variation in viral composition and load as well. The variation in virus composition was based on a *n* = 1 repetition per bee species and *V. destructor* transcriptomes and, consequently, the results do not necessarily reflect variations at the level of species/subspecies and could be due to other factors such as sampling region, season and/or diverse time of sampling of the different bee species and there mites, etc. A higher number of samples in a coordinated effort will be required for species/subspecies comparative purposes.

We found that in the libraries studied the viral reads of Deformed wing virus, one of the most important factors affecting honeybee colony health and survival, were 82.20%, 99.20%, and 97.90% in *A. m. ligustica*, *A. m. syriaca*, and *A. m. intermissa*, respectively, and only 0.76% in *A. cerana*. In addition, we observed that a few viruses of importance were present in *A. mellifera* libraries but not in *A. cerana*’s. Namely, ABPV + IAPV in *A. m. ligustica* and its *V. destructor*, and in *A. m. syriaca*; BeeMLV/VdMLV in all *A. mellifera* libraries and corresponding *V. destructor* mites, as well as in varroa from *A. cerana*; BQCV in all *A. mellifera* and corresponding *V. destructor* and SBV in *A. m. ligustica* and all three libraries of varroa mites parasitizing *A. mellifera*. ABPV seems to be uncommon to *A. cerana* in China and South Korea [41,42], or to show low prevalence in wild colonies [43], as was in *A. cerana* in Northern Thailand [44]. SBV was reported with high prevalence in Southeast Asia [41,45,46,47] as well as BQCV, that showed relatively high prevalence in viral populations of *A. cerana* in China, South Korea, and Vietnam [42,43,48]. These two viruses were absent in our libraries and that could be due also to the small sample size of them.

Furthermore, we identified recently characterized viruses in *A. mellifera* and/or in varroa mites, namely Apis rhabdovirus-1/Bee rahbdovirus-1 (ARV-1/BRV-1) [30,38], Varroa destructor virus-2 and -3 (VDV-2 and VDV-3) [23], and a new Varroa orthomyxovirus-1 (VOV-1). Some of these viruses were identified as common to honeybees and *V. destructor* like ARV-1/BRV-1 and VOV-1, while others appeared to be restricted to mites, such as VDV-2 and VDV-3. Interestingly, VOV-1’s presence was limited to *A. m. ligustica* and *A. m. syriaca* as well as to *V. destructor* mites parasitizing them. VDV-2 was detected in all *V. destructor* libraries and VDV-3 was absent in *Varroa destructor* mites parasitizing *A. cerana*. ARV-1/BRV-1 was found previously in *A. mellifera*, *V. destructor*, and in *Bombus impatiens*, but our finding that it is present in *A. cerana* suggests that it may have a broader host range. *V. destructor* mite parasites of *A. cerana* appear to bear two novel viruses with low homology to Hubei picorna-like virus-29 [39] and to Hubei virga-like virus-14 [40], which were absent in *V. destructor* mites from *A. mellifera*. We validated the presence of a Hubei virga-like virus-14 and designed it VDV-4.

Data suggest that following *V. destructor* invasion there is high selection on DWV strains such that only a single strain seems to dominate, though which strain dominates varies across colonies and studies [20,49,50,51]. This process is accompanied with increase in the collapse of *V. destructor*-infested colonies [15,51,52]. Moreover, laboratory experiments showed that DWV undergoes rapid selection following its injection in the honeybee hemolymph, similarly to what happens during parasitization of *Varroa destructor* on *A. mellifera* [20]. In our study, we measured differences in DWV loads between samples of *A. mellifera* subspecies and their *V. destructor* counterparts. *V. destructor* from *A. m. intermissa* showed lower DWV levels compared to its parasitized host (26.83% and 97.90%, respectively). Interestingly, it was reported that *A. m. intermissa* was more resistant to *V. destructor* parasitization than other *A. mellifera* subspecies [53,54]. Again, the results are subjected to the above-mentioned limitations of the analysis including the *n* = 1 repetitions of the transcriptomic data per subspecies.

We characterized VOV-1, a novel virus common to *A. mellifera* and *V. destructor* and VDV-4, a novel virus of *V. destructor* from *A. cerana*. VOV-1 possess a single-stranded negative-sense RNA genome and belongs to the Orthomyxoviridae family that among others includes the genus Thogotovirus. Most of the Thogotoviruses have been associated with ticks [55] and relatively few of them have been described in Acari or other types of hematophagous arthropods [56,57]. The VOV-1 genome has six segments and this is the first report of the complete genome of a single-stranded negative-sense RNA segmented virus seen in honeybees and varroa mites. We provide evidence that VOV-1 replicates in individual varroa mites, and we found positive sense-virus RNA in pools collected from *A. m. ligustica*. Interestingly, it showed greater prevalence in *V. destructor* mites compared with honeybees of 78.57% and 8.33%, respectively, in samples from apiaries located at the North (Haifa, Kibbutz Lehavot HaBashan, Kibbutz Dan), the Center (ARO, Nitzanei Oz, Herut, Kfar Ruth), and the South (Kibbutz Yad Mordechai) of Israel. This is why, taking together the above data, we decided to name it Varroa orthomyxovirus-1, VOV-1.

The discovery of these novel viruses in *Apis mellifera* and its recently acquired obligatory parasite *Varroa destructor* opens a new venue for investigation of viral interactions in honeybee colonies. A number of questions emerge concerning this new host–pathogen relationship that could interfere the preexisting balance. What is the pathology associated with VOV-1? Do varroa mites transmit VOV-1 and VDV-4 directly, e.g., transovarially, or via their host bee? Where do the viruses accumulate in varroa? Is VOV-1 infectious to *A. mellifera*?

Furthermore, there is another issue concerning those viruses crucial for colony health and survival—DWV, ABPV, IAPV, and CBPV—and their interaction with newly discovered ARV1/ BRV-1, VOV-1, and VDV-2 and -3: Do they affect one another on a mutual base? If they do, on what level and what are the factors that may be involved (e.g., colony location, kind of treatment against varroa, season, colony resistance and/or hygienic behavior, etc.).

Our findings and the tools that we have developed in this study pave the way to investigate these questions and extend our knowledge and understanding of the role played by viral pathogens in honeybee colonies.

## Figures and Tables

**Table 1:** Libraries used in this study.

NGS Libraries	Library Code	Accession Number *
*A. m. ligustica*	IB1	PRJNA329428
*A. m. syriaca*	SB2	PRJNA437728
*A. m. intermissa*	AB3	PRJNA437730
*A. cerana*	BCER	PRJNA475853
*V. destructor* from *A. m. ligustica*	IV4	PRJNA329427
*V. destructor* from *A. m. syriaca*	SV5	PRJNA437729
*V. destructor* from *A. m. intermissa*	AV6	PRJNA437731
*V. destructor* from *A. cerana*	VCER	PRJNA475855

* Libraries uploaded NCBI short read archive database, raw data.

**Table 2:** Accession numbers for the new viruses.

Virus	Accession Numbers
Varroa orthomyxovirus-1 (VOV-1)	MK032465
	MK032466
	MK032467
	MK032468
	MK032469
	MK032470
Varroa destructor virus-4	MK032464

**Table 3:** Presence of viruses in honeybees and *V. destructor* mites by library.

	*A. mellifera ligustica*		*A. mellifera syriaca*		*A. mellifera intermissa*		*A. cerana*	
	Library							
Virus	IB1	IV4	SB2	SV5	AB3	AV6	BCER	VCER
Acute bee paralysis virus (ABPV+IAPV)	+	+	+					
Aphis glycines virus-1 (ApGlV1)	+							
Apis mellifera filamentous virus (AmFV)					+			
Apis rhabdovirus-1/Bee rhabdovirus (ARV-1/BRV-1)	+	+		+		+	+	+
Apis rhabdovirus-2 (ARV-2)	+	+		+		+	+	+
Bee/Varroa destructor Macula-like virus (BeeMLV/ VdMLV)	+	+	+	+	+	+		+
Beihai horseshoe crab virus-1						+		
Black queen cell virus (BQCV)	+	+	+	+	+	+		
Cyclovirus				+		+		+
Deformed wing virus (DWV)	+	+	+	+	+	+	+	+
Varroa orthomyxovirus-1 (VOV-1)	+	+	+	+				
Hubei picorna-like virus-29								+
Hubei virga-like virus-14 (Varroa destructor virus 4, VDV-4)								+
Lake Sinai virus (LSV)	+							
Sacbrood virus (SBV)	+	+		+		+		
Varroa destructor virus-2 (VDV-2)		+		+		+		+
Varroa destructor virus-3 (VDV-3)		+		+		+		

Determined from the percentage of total viral reads mapping to viral contigs of the honeybees (IB1, SB2, AB3, and BCER) and *V. destructor* mites (IV4, SV5, AV6, and VCER) libraries with a cutoff at 0.0001%. BLASTX against Genbank, NCBI.

**Table 4:** Comparison of the Varroa orthomyxovirus-1 (VOV-1) contigs to the Orthomyxoviruses Thogoto and Dhori (THOV and DHOV).

VOV-1 Segments	Length of Contig (na)	Similarity to THOV/DHOV	THOV/DHOV Proteins
1	2198	58%	PB2 (DHOV)
2	1899	29%	PB1 (DHOV)
3	358	46%	PA (DHOV)
4	232	41%	GP (THOV)
5	1442	39%	NP (DHOV)
6	983	23%	M (THOV)

**Table 5:** Genomic copies of VOV-1 in individual mites and in pools of mites and nurse honeybees.

Location	Hive #	Sample Type	N	VOV-1 Genomic Copies
ARO (C)	7	Mite	1	3.54 × 10^5^
ARO (C)	13	Mite	1	1.22 × 10^6^
ARO (C)	19	Mite	1	4.43 × 10^5^
ARO (C)	81	Mite	1	9.40 × 10^4^
ARO (C)	7	Mite	1	5.34 × 10^5^
ARO (C)	401	Mite	1	6.32 × 10^5^
ARO (C)	401	Mite	1	2.50 × 10^5^
ARO (C)	6	Mite	1	4.23 × 10^5^
ARO (C)	7	Mite	1	2.69 × 10^5^
ARO (C)	13	Mite	1	4.00 × 10^5^
ARO (C)	13	Mite	1	1.83 × 10^4^
ARO (C)	19	Mite	1	4.62 × 10^5^
ARO (C)	19	Mites	1	5.11 × 10^2^
ARO (C)	11	Mites	1	4.19 × 10^5^
ARO (C)	9	Mites (p)	6	3.27 × 10^4^
ARO (C)	10	Mites (p)	6	2.85 × 10^4^
ARO (C)	7	Mites (p)	6	2.95 × 10^4^
ARO (C)	38	Mites (p)	6	2.01 × 10^4^
ARO (C)	401	Mites (p)	6	1.44 × 10^4^
Nitzanei Oz (C)	1	Mites(p)	6	2.73 × 10^4^
Haifa (N)	1	Mite (p)	6	1.35 × 10^4^
Lehavot Habashan (N)	1	Mite (p)	6	1.64 × 10^5^
Lehavot Habashan (N)	2	Mites (p)	6	4.88 × 10^5^
Lehavot Habashan (N)	4	Mites (p)	6	2.33 × 10^3^
ARO (C)	MIX	Mites (p)	6	3.08 × 10^5^
Kfar Rut (C)	17	Bees (p)	10	2.01 × 10^4^
Dan (N)	14	Bees (p)	10	4.91 × 10^2^
Kfar Rut (S)	43	Bees (p)	10	1.38 × 10^5^
Yad Mordehai (S)	13	Bees (p)	10	1.25 × 10^3^
Yad Mordehai (S)	36	Bees (p)	10	9.99 × 10^4^

Quantitation of VOV-1 genomic copy number was carried out by amplifying segment 6 using specific primers (see Materials and Methods). N = North, C = Center, and S = South of Israel, respectively. MIX, group of mites from various colonies. (p), pool.

## Supplementary Materials

The following are available online at http://www.mdpi.com/1999-4915/11/2/94/s1, Figure S1: title, Table S1: Table S1. Primers used for validation and quantitation of Varroa orthomyxovirus-1 (VOV-1). Table S2. Primers used for validation and quantitation of Varroa destructor virus-4 (VDV-4). Table S3. Complete VOV-1 nucleotide sequences. Table S4. Nucleotide sequence of VDV-4.
