# Supplementary material for: New Viruses from the Ectoparasite Mite Varroa destructor Infesting Apis mellifera and Apis cerana
Source: Viruses. 2019 Jan 24;11(2):94. doi: 10.3390/v11020094 (PMC6409542; doi:10.3390/v11020094)
Supplement: Supplementary file 1 [file viruses-11-00094-s001.pdf]

Supplementary material

Table S1. Primers used for validation and quantitation of Varroa orthomyxovirus-1 (VOV-1)

Name*	Genome segment	Expected size (bp)	Sequence	Application
VOV1-140F	1	2001	GCGATGAACGAGAGATTGGC	diagnostics
VOV1-2141R	1		AGTCGGATTTCTTTTCGCCG	diagnostics
VOV2-184F	2	1704	TCAGGGTACGCTTGTTCCAG	diagnostics
VOV2-1888R	2		TGGCAAAGGGAAGACAAACA	diagnostics
VOV3-63F	3	242	ACCGTCGTATATTGGCAAGGAA	diagnostics
VOV3-305R	3		TGATACAACCGTCATCAGCTT	diagnostics
VOV4-21F	4	1626	TCTATCATGGGGAAAGGATTGG	diagnostics
VOV4-1647R	4		ACACCGCCCATACATTGACT	diagnostics
VOV5-58F	5	1336	AATGAGAGCATGGAAGTGGCT	diagnostics
VOV5-1394R	5		CACTGTTCTCGGGGGTTAGAC	diagnostics
VOV6-46F	6	824	ATACTCTGGCAGTTGTGACC	diagnostics
VOV6-870R	6		TGACATTCAAGAGAGGGCCAA	diagnostics replication
VOV-1-qRT-F1	6	189	ACTCCCTTCAGGTACAGGCT	qPCR
VOV-1-qRT-F1	6		TGCACGGATTAGCCATTGGT	qPCR
VOV6-46FTAG	6	840	AGCCTGCGCACCGTGGATAC TCTGGCAGTTGTGACC	replication
TAG			AGCCTGCGCACCGTGG	replication

*The numbers after the hyphen indicate the binding site of the first nucleotide of the primer in the viral genome.
Suffix F and R indicate orientations forward and reverse in respect to the viral genome.

Table S2. Primers used for validation and quantitation of Varroa destructor virus-4 (VDV-4)

Name*	Expected size (bp)	Sequence	Application
VDV4-1451F	1438	CTGTAGCTGATACTGGGGCG	diagnostics

VDV4-2889R		ACTACGACCCTCGTCATCCA	diagnostics
VDV4-6242F	1576	CTGTACCTGAGTTGCAGGGG	diagnostics
VDV4-7818R		AGCCAATTCCAACGGTCCAT	diagnostics

*The numbers after the hyphen indicate the binding site of the first nucleotide of the primer in the viral genome.
Suffix F and R indicate orientations forward and reverse in respect to the viral genome.

Table S3. Complete VOV-1 consensus nucleotide sequences

VOV-1 segment 1 - 2198 nucleotides

ACCTTTAAGAAGTACACATCAAGTAGGAGAGAAGATGCACCTCATGCAAGACTATTTTACAATGTCA
GGAAGCCATGGCCACTTGTTTGCAGTAATTACAAAAACATTCCAAAAAAGTTTGGTGGTGAACAATT
ATTAAGCGATGAACGAGAGATTGGCGAGCATGACAGTGTGCGTGCATCAATACTGACAGCTGACTA
CTTGTTCAAATATGGCAAACCTGCAACAAAGGAAGTTGTTGATGCGTTCATGGTAGTAGAGAGAAAC
AGAGTGAATAAGAGATTCAGTGTGGATCTTGGGAATATAAGGTTTGACTCGTATTTACCAATAAGGA
AATTGGTACATTTTGAACCTATTATACCCAATGTCCCTCAAGCATGTGTAAACCCTACGATAATGAAA
GCTCTATTTTCGGATCTAGCTCCAGTCGACCTGATATACCAGTCCATCCCTGACAAAGTCCGTGAGCT
CCAGCGAATCCTCCATCCGCTTATGCAGTATCGTAGCATGGCAAGCACTGTACATTTAGCCCGTTCTT
GTATAACACCCATTAGTAGATGGTTGCCTGTTATAGCTGACACGTCAAATACTATTAACGAGTTATCT
TATTTCTTGTACAGTAATTACCATCAAGTGGAAGTAAATGAAGACCTTATTGCTACACATGGTGGAGT
AGAGAGGTTGTGTTCTGAAATCATCAAGCACGCATTGGCTTACAAGGATAGGGCTAAAGAAAAGTG
TTTAGAGTTTTGTAATAGTGTGCATGTTACAGGGGTTAGTTTAGAGTCGGTGGTGGAGGAAGTTAGG
AATGAAGGGCCTTTTACTAATATAATTCGTTCATTATTAGGATACCCAATAAAGAGCGGGAAGACTAT
AAGAAGAACTAAGTTTGAGTTGTTAGATTCAGAGTACCCTAGGACGCCAATTACTATAAAGAAAAGA
TTTATGGGAATAGAATGGGAACATACATTTGAATGTTTTACAGGCTTTTGGAAGGTCTACTTTGCTAA
TAGAGGGATTAGAGGGTATGCCCAAGGATCCAGTAGAGTGGTGAATGGCATATGGTTATTGGCAGA
AAAGGGTACAGATATTATAGATGCAATGATTGATCTACACAAGTATGTAGCAGCTGTACATACTGGG
TTTGAGGGAGATTTCCCTTCTTTTGTTTCAAAGAAAGAGCATTGGGAGAAATTCTATACGTACCACAA
GGATAATCCTATAGAACTATATCGATCATTAGGCATCACAAACCATGGTTATGTGTCTAACTCATTCA
TTGTCGGTGAAAAGAGTAAGGGTTATTTGCAGAAAGTAAGGCAACAGACGGAAAATGTTCAACCGT
ATACAACAAGTAATTATCATACTGAAGTCGACAATAGGCTAAAAGTGTACATCAAGAAACCTATGTA
TACTGAGTTAATCTATGATCCGGAAGAAATTCCTTTCTATAGGAACGCACCCCTACCCACCGGACTTT
CCGTTACTGCGCCTGAACTCATGTCCATGCATAGAAGTGCACTCATGCAGTTGGAGTTTATGGTTAAG
GATTACATCCCCGACAAACACAAAATGCAAGAATTGGTAGCTGCAACTCTTTCTGAGGCAGAGACAC
CGATCACTTCTGCGTTCAAAAGCATAATAAAACCCCAGCCTTCCACCTCTGTAATGAAAAGGAAATTG
AGCAGTTACTTAGAGGCAAACTTGGACTGGGCGGAAACCCCTGCAAAGAGGGTAAAAGCATTCTAC
TACGTGTGTGGGTCTAATGCTCCCCAGCAGAAGACAAGAAAAAGGAGATACGATGAAACTACATTG
TCCCTGATTGACGATACAGGATTCATTGAACCATCACTCAAGAGAGGAGTCCTTACAGCTAACAAGA
ATACTGTAAAATTCCTCGGGAAGCCAGTAACACATACGTCATGGGGCACTAATTCTGAGTACATTGCT
GTATGTTATACCACTATGAAACCAGAATTTTTGGTTTGCTCTAGTATGGAGGATGCACATAACCGTAG
TTTGACAAGATTTTGTATGCAGTCCTCTGGTTGTTGGATTACATTCGAGAAGAAAGAGGCTAGATGG
ACTGAAGCAGAGAATATTAGAAAAGCACTTGATAACCGGCGAAAAGAAATCCGACTGTTTGAAGGC
GAAAGCAGACTTCTAAAAATGATGTCTCAATAGTTTGGTACCTGTAA

VOV-1 segment 2 - 1899 nucleotides
TTCAGCACAAAATAATGATAACGGTTACAATGTAATTTATGAATTCCACACTAAGAGTCTAGTTTCTCT
CTTAACAACTTCTTGGTTTCTTCTGGCAAATTGGATCTTTCAATAAGGGTAAAAATCCGGTTATACAA
AGCTTCATTAACTGTACCAGGTATACCAACATTAGACACCAATAATTCAGGGTACGCTTGTTCCAGAG
CACCACACATTTGCTGATACTCTTCCTCTTTTTTCAGCTGATTTCGGTTAGGTCTATTCAATATGGTCCT
ATTCCGTGTAAACTGGTATTTATAAATTGAACCAACCGAGTTGTCTTCAATGACCACAGGCATTTTGT
TTTCTGGTCGAAATGACACCATAGGTTCATTAGTCTGCACAAGTGAATTCTCAGGATTGAGCAGGAT
ACTTATTTGTCTTGCATCTAGTAGGCCCAGATGCTCTCTTAATGCTATCTCATCTAAGTGGAGTGTAGA
TACACTGTGTACCGATTTTGCACCCTGATTAGTTAGTAACACTGGTAAATCATTTTCCAAAAAGAATCT
TGTTCTTTTAGTTTGCCCCATGGCTAGATAAGCATATTGATATGCTTTAATGAAGATTCTTAGACATAA
AGAAATGGTACCAATGCACATTTGCCCAGTATTGAAAGAATGCTTCAATACACTCAAGCCCATCGCTA
GATCGGTACTAGGATTTTTACCGCTAGGTACTAATGATGGTAGATCTGTAGCCACGTTTCCAACAAAA
TCTCCATTGTGATACTTGGAATTGAATTCACCTGTTCCAGCAGGGACTATTAGCACGCATTTACTAGG
TGACATATTTACTCCAACGAGTTTGAAATTAAGTCTCAGTGTTTCTGCTTGGCTAAACAAGGTGTCCA
TATCTTTAACATAAAACCAATGAATGAAGTCATCACTGCTTTCAACATGATTACCCGGTAATTCGGGT
CTATCAGCGGCTATGAGGGCCAATAGGGTAGATGTTAAGTTGTACATCCCCATGAACATTCCAAGAG
TACATGATATATTTTGACCATGTAATTTCTCTTTGGGCACCAGTAATACTTTCAAATAATCATATTCAG
ATTCAAGTTCACCAAAAGGGAAGACCTTTGTCATTTTACCACATTGATATGTTAATCCCTCCCCTAAAT
CTGCTACTTTACTTTTGAACAATAAGAAGGGTACATTAAACAATTCAATCATCCATTCCTCAATTCCGG
TCTCTTGAAGAAACACTGTCCACATAAGTCTCATGGCGTCTGGATCCAAACACTCATTAAACTTTTCTT
GGTCACCACTTAATTCTCCAGTAACAGGAGAACGGTCTGAACTTAGTTTGCTCTGTAACTTTGCCAGC
TTTTCTTCACCTCCTACTGGGAGACCACTACTATCTATGTCTTCCAATATTACAGTAGCAGCATCTTCA
ACCAACTTTACAAATCCTCTTAAAAGCATGCTTGGCGTTGCTATGGTCCTCCCATTTAGCCGTCCTCTC
TCGAAATGTTTCCACATGGTTCCCAACCTAGAAGCATGAAGAAGCAACGACTCCTGTGTTATTATTGT
TATCTTCTTGACCACTTTCTCCTGTGGCTGTCTCTTCATAACCTTAGAAGGCCCAGGCTTTTCTTCATCA
TCAGACTCTTCTTCACTAGTGGTGGTCTCATCATCTGAAATTTTCCCTTTTTTGTAAAACTTTTGTTCCT
TTCTAGTTGTCTCCATTGCCAGTACATCTATTACTGCTTGCAGGTAAGAGAGGAGACTTTTCCCAATG
CGATACCCTAGATTAGACTCTAAAAATTCTACAGTTTCATCTAGGGCCATCGCTGCAGGAACGTTTCT
TTCTTGTATGAATGAGTATGTTTGTCTTCCCTTTGCCAAGACATTATAG

VOV-1 segment 3 – 1981 nucleotides
GTTCATTAATTCTGAATACACATATTATACATAATAAATATCGGGGAGATCATTCCATGTTTTTGCCAA
ATATAGTACTATGGGGTTATTTATAATACATTCTTGACATTTAGATTCTGGGCTTGATCCATATGGAAT
AAATACACCTTTCTTCAATACCATAGCTTGTCTACACATATGTAACCGTCGTATATTGGCAAGGAATCC
TTCCATTTGGTTGTCATTCCATATTGCCATTAAAAGATGCATAGATAGCATTTTAGTTGCCCATATTAG
AGCACTCATCCTTGCTGCCACGAGTTTTGTATGTTCCTCGTCGGAATAAATAAAAAGCTCAACTTGAA
CATCCTCGGAAAGATCCATCTTTCCCTTAAGAACAGCATTGTTCATCACAGTTTTAGAAAAAGCTGAT
GACGGTTGTATCATACATTTTCGCATAGCCATGAAATAGAAAAGTCTATACTTGGCTATAGATGTACT
TCTCAGCAAAAATGGCTGGACTGATTGAGGGTCTTTTGCATCTATATATTTGAAATACCCATATGCAT
GGTTGGGGTATTTTCTTGGATTGTCAGACCCAACAAATTCTAACACAACCAAAGGTACCCTATCTGAA
TCGTGCTTCAGATGGTGTGGTCCCAAACACACAAACCCCCACAACTGTGTCATAGGGGTACCATATAT
ACATCTCCGTGTAGAAATCGGTAGGAATGTTACTTTGTTTCTGTCAGTATTGAGCTCTTGGTAGACCT
TGGTTGAGGCAGTTACCCACTTTTCCATCACAGCTGCTGCCCATGTACATGTAACTGCCTCTAGGAAC
TTGTCTGCTATGTCTTCAGATATCTCATCCAGCTCGCAATGTGTATATGGTCTTTCAAGCTCAATCCAT
TCGACAGAATCTTTTTTATCAAGATCGTTCAGCTCCTCAGGGACCCATTTTGGCATAGACTTATGCTCA
ATGGGGTAATCAGCGCGCTTCCATTCTTCTTCTGAAGGTGTGCCATAGAGTTGCTCGTACTTTATCTTT
TTCTTCCTTTTCAAGTTGACTCCAAAGCTTTTAATATCACAGGACTCGATATGGCGCCTGTTGCCACCA

TCTGCATAGAACTTTAGACTAGTCATAATGGTTTGGACCTCATCATTGCCGGTGTGGCCTTGTCTTTTA
AGCACCACAGTTTCAACCCTATCGCTGATCTCTTTGCATACGACCTGGTCACTTGCAAACTCACTCATT
GTATACCTAGAGTCGCAAGCAATCAAGAAGGGCTTCCAATGCTGTGGTTGTTTGTCCTCATTGAGGT
CAAATTTCGGTAGTTTCTTCATCTGTGTCCAAGGCTTACCCAACAATGTGCTGTCCTCCCCTTTTGTTG
TAGTGTCTGGGATAAATGTCAAATCACTATCTTTAATCAAAAGCCCAGCTAGGAGTTTTGGGATGGT
CACATGAGTACTATGGCCTGTAGAGAGGACCAGATCTAATAGCTGCTCTTGGTTTTGTGAAGAAAAC
CATTGCGTACATTTCACCATGCAACTCGAACAAAGCTGGGATTGGGCCTTGTCTATAACTCCGGGGG
AAGAGACATTTAATCTTCCTGTCTTATCTATTACTATAAGAACTTCATGGTCTCTAATTGGAGAGTGTG
GTCTGCTTCGGTTGATAGAGATATTACTAATCTAACTCTTATTACCACGCGTAGCTTTTTATCTAAAAG
CCCCCCTTCAGCTAATTGTCTGACAGAGACCCCGTTCTCTTCTGCAATCTGTTCGGCAAACTCTTTGTT
ATGCCATTTCAGAAATATGTATCTTGACATATTTCTATGTTTGTGGTCATTACTGATCTGGCAATAAAT
CATGTACTTTAAACATTCGATTACCCTTCTGGTGTACCCATCGCTAATGGAATTCCAGTCGGCTTGTGT
TTCTGATAATTGCCATACCTCATTGGCCACGAATGCAGGTGCTTGTACTTCCATTTGGTTTGAT

VOV-1 segment 4 – 1708 nucleotides
TTTTTTTTTTTAAATATCTCTCTATCATGGGGAAAGGATTGGTATTACCATATTACTTTAAGCAATAAA
AGAAATAATAAAGAACAGAGAAGTGCACTCACAAATGATACTGATGTCAATGACAAAATACTGCCGC
ACCAGGTGTTTAGGGGATTTTCCAAAACATCGGACATCAGTGCCGAACTATACTTTTTAACTCGGTTC
ATTGATTCTTCCATGTTTAATCTTTCTAGCTCTATAAATTCGTAGATACTCCATTGAAAAGAGGCCCCC
TCTATAGATACTCCTTTCTCCAGTCCTTCAATTTCATGGATTTCATGTAAAAGGACGATGGGAGTGAC
TTCTGCATTTGGTTTGAGACATACACTTTTTCCATCCCAGGGGACAGGGATTCCACTCTGCATAGTAA
GCCCCTGAGGTACACAAAATTCACTCACGGGTTTGTACGGTCTGGCAATAAGGCATTTAGTGAATAG
ACCCCCTGATGTATATACAGTCCGGTATAGTTCCTCACCCTGGAGAACTTTACTCAACAATAGGGGGT
CCTTTTGACTGAGATTTTCAATTAACCGAGTTACTACACCCGTTAATTCGGTAAGACCTTTTGTGAGCT
CAAAATCATTCAAATCACTTCGAAGTGAGACTTCCCGAACTAGCGAGTCAACCTCATACAAATCACTG
AGTGATGCACTTTTGTCAGATATAATAACTCTCTCTGAAGCTGTCATATTTAAAGAAGTTAAATTTACT
AAAATGTTACTATGACGATTTGCGACGTATGTAGAATTCCCAGAATAGACACCACCGATCAATTCTTT
AGGACCTGACCTCAGAATGCATTTTAATCCACCGGAATTTTCTGAGCCCAAACAATCCAAGATCACTT
CAGATTGATGCTTTAGAGGGAGTGTGGGAATATAGAGTTGAGCCCCTTTCTCCATTGTATAAATATG
TTTCGTGAGCTCAAACATGGTACCGTTGCTGAAAGACGAATAGATCTTACCATCCTCTAGGACTCTAA
CTGGAAGATCAATTTCTCTAATAAAACATGACCAACTGATTAGGCATGTATGGTAAGGGAACTTAAT
CCACTCCCCTCCACTACATTCATGCCTTACCTCATCATTGACCCATTGTGGGTTGGTAGTCAACAAATC
TCTTGGTGACTTAAGGCATTCTTGTGACCACGATCCCCAAAAATCGCTCTCTCCACAGAGATTTCTCCC
AACTTGGCATTTTTTGGTTCTAATCCACTCCTCCAGTTCACTGGTATCAGGAAGAAATGACTGCCATCT
TTTCATTGGATAGGAATTTGTGTCTAAGGCAGTTCCATAGTATTGGTAATTAGTGTACAGTTCTCTTTT
ATATAGATATCCCTGCACCGTATGATCATACCAAGAGAGCATTCCAACTGCAGTTTGTTCCTTGATCA
TCGTCTTAGGAGGCATTGGGGGTTGAAGTTTGAAGATACCTCGACAATCCACCCCAGAGGAATCAGA
AGAGTCTTGTTTGGGATAAGCATAAGAGTATCTGCGATAGTGCTCGAGGTGTGACAGAGTGACTAT
GGATACGATAACACTCATCCAGTAGAGATACGTCTCAAACAGACCCATTGAGTACATGATTGAGTCA
ATGTATGGGCGGTGTGTTCTTCAATCCTTATGTCTGAGAGTGTGCGCGTGGTTGTAGGTGAGGATGA
CTAGCTAAT

VOV-1 segment 5 – 1442 nucleotides
GGAGATTACATTATATACTATATATGTACGATTGAAAAGAGAGTTTCTAACAAAATGAATGAGAGCA
TGGAAGTGGCTGTTGGTACAAGTTCTAACAGAGATGAACTTGTGACAAGATTCGAAGCGAACCCCA
ATGATATACGTGATAAATATAAAGAGTACCAAAGGTTGATGCTTGCGATGGCGAGTACAGCAGACA
TAGATATAACCATAGATAGAAATGCAGAATTGATCGGAAGTGCAGTAATGGCAGGGTTAACAGAAA
ACAAGAGCGTAGAACGAGAATTTGAAGCAGCCTTTGTAGTAGTGAACAGTGCAGGAGTGATAACTC

TAGTTGACAAGAAGATCTCAATGGATGTGGTGAATTCAGCACGAAATGTGAAGCTCAATACCCATCA
GGGAAGGGCCAGTTGGTATCCATTCTTAGCAGCTTTACAATTGTCAGCCAAGACCAAGGACCAAATC
CTGTGGCAGAAAAGCAAGGTCACTACTGATCTAGGAGTCCCTACTGTGTGTGAACCCTATGCAGGTG
GTTGGCACATCAAGGACAAGTTCAAAAGGAGCCGAGCATTGTCAATAGGCCCAATAACCCATCTTTG
GATGTATAAGAAATTTCAAGACAGACAGGATGGTAGTAGGAAGAAACGATTGGCTAAAGAAGCTTT
ACGAGGAATCAAAGAAAGAATTCGGAAAACTCTTAAGAGACAAAGCATTGGAGTGACTCAAAAGAA
AATCATAGATGCTATATTTGACGAAGACAAATGGGAATTAGCAAAAACCCTTTGTTTGAGCTATTTAG
GTATCAAACCACACATTGAACATCATTTTGTGATGACTTATCCGTTAATAGCAGTTATCAATGATTGG
GAGGGAGCTAATTTCTCTAATGAATGGGTCTGGATTACGTTGTGCAACAACATCCAGAAGATATCAT
TTGCGGCACCAGACAACACTTGGCCAGAGTTTTTAATGCAATGTAACATCCACGGAGTGTTACAATC
GCAGGCTGAGGATCTTGGATTGCTAGAAGACATTTTTGGCATGAGATTTTACCAAAGAAAAGATTTT
GGTAGGTTTGACAATAAAAAGGTAACCTTGGTCTCCAAACCAGCAAATCCGCTTGCTTACAAGTATT
GGGTAAGACCTCAGAAAGGCGCACCTAGAGTTATTGAAGGGGCTAGACGTGGTCAAATTTCCTCTA
AACCTTCACTCAAGTGTGCACGGCGGTCATACAATCAATTTACTAGCTTAGATGAACTGGAGAAAGC
TTACGTATCAGTCTCCAGTGAGAATTTTGTTGAAGAGATTAACAAAGAATTCTCAGCTTATACTGCTT
TACAGTTGGAAGGATCTACTTACGCTTTTAAGAAAGGGTCTAACCCCCGAGAACAGTGGCCAGGTCA
AGTTGCTACAAATGGAAAGTACCTCTTTGGTAATTGACG

VOV-1 segment 6 – 951 nucleotides
TATAAATCGGAAAATAATTTCAGAAAATGCAGAAAAAATGCAAGTATACTCTGGCAGTTGTGACCAG
TCATTCAGATCTGTATAAGGATCCTCAAACAATGAGATCACAATGAACTCCTTATAGCGTTTAGTCTA
TCAAAAGAATTCTTGATGACTGAGTACGCTATTCCTTTCAAGTTGAGATAATCAGGACTATTAGCAAT
GGAAAGCATGGCATCAATCCATCCCATGTAAATAATGTTTTTATCATAGGACTGCTCTGCATTTCCAA
TGCTTTTGACCAGTCTATGTAGCTCTCTATTGGCATCGTGTATAGCAGTGACTGCGCTGGACACAGGC
TTTGCTTTAAGTAGTGGTTCAATAACACAATCTAATGTATTACTTATTTTAATACACTCGCTTATCCAAT
GGTCATCATATGTCTCACACTGTTCCTCTTCAATGCTACTGTTACTAACTAAAGTAGCAAGTTGATCAT
CGTGGCTTGGTTTCATGTTATTAAGTAAATTGAGCAGTTCAAGGAGTTGTACTAGATACAACGGTGT
CTTAACATCCTTTACGATCTCGTAGCATAAAGCAGGAATATTGGCAGGATGCACTCCCTTCAGGTACA
GGCTCCTCCCAGCTTCAGAAGCTTGTGACTTGTATCCTTGATAGAGTAGTTCCCCTGAGGTACCAACA
GTAGCGACAGATTGTTTCAAAGACCAAAATCCTTCGGGCATACCTGCAAACAAATGCTTATACACAA
GTGCTAGTTCCATTTTACTACCAATGGCTAATCCGTGCATATCCTTAACCATCTTCCATTCTCTATTCGA
AAGGTGGCTGTCTCTGGTCATAACCATAGACTTGGCCCTCTCTTGAATGTCAATAGAGCAATAACACC
AGATCTGCTTTTGCGAGGTAGCCATTGCCATTGTTTCCATGTGGTTTAATTTTTTATAATGTTAG

Table S4. Nucleotide sequence of VDV-4
CTGACTATCGATATCTACCTTAACATAAATGTGATATGACTTAATTTACCCGTTGGGTCA
TTGCTTTTATCAATAGCGTGTGGGGAGGTACCTCTTGGTACTGTTCTCTACTTCGTGTCT
GTGAGTTATTCTTACCGGCGAAACTTAAAATTTTATCTCACTATGGCTTTTCGTAAGAAA
GTGTGTGAGATTAAGGAGGTTCTGACTGTTAGTCAGCAAGAGCTTCTTAGTTCGATGTAT
CCTGAATATGATCTGGTTTTTAAGAATCTACAGGTCAATGATCATTCTATGGCTGCGGCT
AGCCGTAGAATTGAGACCCTTGTTTGCTTGGACAAATTGGGGTACGATTCTTTTAGGGGC
GTGCCAGATGGCTACGATGATTTGGTAGCTGATTTTGGTGGAAATTTTATGTCACATTAT
TTTTCTAACCGTTCTAATGTTCATAGTGATTGCCCTGTAATGGATGATAGGGATAGGCAA
AGGTGGGTCAATAGGATTGAAAGTATTTATAGGAATTCTGTCAAAGAAAATGAGCGTTGT
TCCCCTGAGGAATTCCATAAGAGTTTTAAAAATAGATTTGCTCATTTAGTCTGTGACAAG
GCTAGTCAGGAATGTCGTAGGCGAGCTAGGTATGGTTTGATGATACATTCGGGCTATGAT

TTCAACGTTCGTGATGTTTTTGAGGCCATGATTAGTCGGGGTATTATCGAACTGTATGGG
TCCATTATTTTTGACGATCAGGTTATGTATTTAAGAAAAGGAAAAATCAACTCAATCGAA
AGTAGATACACCATCACTGACGACGGATATATAAAGTTTTCTTTTAAAAATGATAATAGT
ATGGTTTATAAACATAGGTTCGTGGATTATATGTCATATTTTTTACTTACTCAGGTAGAT
TTTCCAAAATTGGCTAAACGAGGTATTATAGAATTACTTGAAAATAGGAATTCGATTCAA
TTTTATAAATTGACGGTTGTTGACGTTGATGGTGAGAATGGTTGTTCTAGGGATATTCTC
TTTAGGGATATAGCATTGCGTTCGCTTGAAGGGAAGACAAAAGTAAAATTTTATACTTAT
GATTCTGACATGTTAGTTAGGTACGGACCTAAGAAAGCCATGCAGGAAGTTATTATGTAT
GCTCCAACTGACATTGTAGACAAAGCTATGGCTCATGCCTTTTCTATCACCGAAGTAAAA
TTTCGACCAGTTGAAGTTTTTAATTTTATAAGATCTTACGAAGGACGAATTTTTTTTGGA
AATGATGTATGTGTTAGAACCAAGTCTCAGTCATCAGAAAAATCTTTTATTTTGGCTAAT
GCTATTTATGTTCATGCGTATATAAAGAAATATAACGTGGGCAAAACTGTTCAGAGTTTG
CTTGGTGACATTTCTTATATTAGGGAGGCTAAATTGGGAGGTTGGATAACTAAGTTTAAG
AAATTGTTTGCTGTAGCTGATACTGGGGCGTGTGTTTTTAGTGCGGATGGAAAATTTTCT
TACGGTCTTATTAATTATATTTTGTATAAGATGCTAAGAAATAGAGCCGTGTCACAAGAT
GTCGAGTTTATAATGACTGTGCCTGAGTTTATTACGGTAGGTGGTGCGACTGTCGGTTCC
ATTGATCAAGAATATAACGAGACCTTTTCGCAATACGTTTTCGGAGGTGATAGTAAAAAA
TATGATGATTTTATAAAAGAGTTGAATTCGGTTTATTCGGGTAACAAGGTACTTTTAAAA
AATTTAGGAACTGGTGGAGATAATATGATTTGCGAAAATTATTCTGTTGGTTTCGATGAA
GAACAGTATAGTTTCGTTGTTCGGTTGTCAAAATTTTCTTGTATTGACAGTCGTAAAGTG
TTGGCTGGTTTAAGTTCTAGGGTGGATTTTATTCTTGGAGCTGGTGTAGGGGATTATTAT
GATAATTTTCCTGGTGTGTATTTTTCGGGAAGAAGGGACAATGGTATTGCTCGTTCATAT
TATGGTTCTATTGGCAGCACTAGGCAGCTCAGGTCGATCGTTAGCAAGGGATTGCTTATC
TTTTCAGATGCTGATAGTGATACGCTTGGATTGGCTTGTAAATTTTTACTAGAATTTAAT
ATTTTTTATAAAGTTCCAGTTTACTTGTATGTTAACTCTATACCCGTTGACAATTACAGG
GTTTTATCGAATCTTATTGGTAGGTTTAAGCGGGTTGGTAATTGTCCTCATTTGTATGTT
TTAAAGGGAAGATCATCATGTCGTCCTGATTTATCATGTGGTTATTTCCGATGCGTAGAT
TATTATTCTTATTATAGCGCGGTATTGGGTGTTAATGAGGTGAATGGTATAAGTTTGTTT
GATAACATGAGATCATATTTGCTTCCGTTGGAAGTTTTGACTCTCATTGGTCGGTTTTAT
CAATTTTCTGGACGTAGTTTGTATGTGTATAACAGTATAACTGGAATTATACCTAGGTTT
TTCTTTGAAAATTATCAGGTATATTCTTTCTTGCAGGTAACTTCTAGGGAGAGTTTCGTT
ATACATGATGTAAATCTTAAAGAAACTCCACATGAGAAGTTTTTGGTAGAGAATGGACGT
GTGGAGTGTGCAGGTTATTCTTATGTTTTTATAAATCTTGTTGATGGTATTACAGATTTT
GATTATAAGGTTTTTCATAATATTTTTGATGGGAAGTTGGAGAGGATGGTTGTACTTACT
TTGCGATGTTTTCCATATTATCCTCCGGATGGTTACAGTGGGTTTTCCTTGGGTTACGAA
ATAGATGCTAAAGACTATGTTCTTACCTTTTACTATCGAAACGATCTTGATGTTTCCGTT
GGAGATGTTGTTATTTCGGTGGGTTATGAGAGTGAAAATAGTGTGGAGATGGATGACGAG
GGTCGTAGTGACACTGAGATTGGAGATGGAGAGGTTTTACCTGTCTTGACACAGGATGTT
GACAGTTCGACTGAAAGTGTTGCGGAGGACCTGCAGTCTGGGGCATCGTCGATATTAATT
GATGAAAGTGGATGTGGTGGAGGTATACATGTTGGGACAATTGAGGAGCGCTCCATATCT
TCCGTGGGATATGTTAAGAAGGAGGTTCGGGGTGATGGAAATTGTCTTTTTTACGCTGTT
CTCGGGTGCAGTGATCATGATAGAGCCTTGCGTTTGAGAGAGCAACTGCGGGGTTTTGGA
TCGATGGTTTCCGATGCTTCTTTCGTTTATGCGGATCTATTGGAAGAGACGGCGGCTGAT
GGTGTTTTTGCTGGTACTGCGTCTATTATAGCAATGCATTATATGCTTAATCTTGATTTT
ACGGTCTATGACGTGGATGATTTTGGTTTGCGCAGGGAGGTGTTGGTCAAAAAGAGTCAG
TATCCGTCTGGACATATTTTTATTGAGAGATGTAGACAACATTATTCGTATTACCTTAGG
ACTTGTTCAGGTGAGGCGTGTGACATGGTTTTACCTGTTGTATTACCGGATGTTGGACAG
GTTAGGTCTGATTTCAAAAAATATATGGGTATTCCATGTGATAGGTATTCCTGGCTAAAA

AGGTTTTTGGGTTTGGGTGTTGAGGAGTTTTCTGATTATGGAAAAGGTGGAAAGGTGCAG
TTTGTAAGTTTGCTTACTACTGAATGTGCCTCTTTGAAGTGTACTTGCTTGCATGGTATT
CGTGAGTATGTTGATGGTACTGGATTTTCTATTGCTGTTTTGCTTAGACTTAGACAGATC
GAGAATTCGGACATTGATGGATTGCTTGCTGATAGAGGATTGTCTTGGAGTTTGCTCTCG
TTGCCGTCGGTTGCGGTTGCTGAGTGTATTATTTTGCTGATTACAGATTTGCAATCACCC
GCTGTTGGTGGCGATTTGTCCGAGTCCGTCGCCAGGTACAGACAACATAGATCGGAGTGT
GATAACTGTGAGCGTGGTGTGTTGGGTGAAACTAGTTTTGACCCCTTTTATAGGACGTAT
TATTCTTGCTGTGACAAACCAGGTAATGTTATTACTCGTTCGAGTAATTTAGATTCGTGT
AATCGGGTTCTTAAGGTAGATATCGATAGTCAGGTTAGTGGGGGTTATAAATTTCTTGAT
AATGATAGGACGGTATATTTAAAGATATCTACTGAAAGTATGAGAGATGAGAAGATTTTA
AATGGCGTTGAAACGTACATTGCTGAGAAATTTAAAGTGTTTGGTATGTCAGTGGTAGGT
TTGGATGTTGAGGATGGGTATACTGATTATTTTTCTCGGATTTTGGCGCAATATGCTGTT
GGGGTCATGGTTTTGGAGAAAGATGTGGGTACGCATTTACCGCAATCTATAATGTCTTCA
ATTGGATTTCTTATAGGAGATGGAGAAAGGGAGTATAGGATCAATTCCATGCTTGAGTCT
AAGGCTTATTATCAAATTCAGATTGTTGATACTAAGGAGTCGCTTAGGGTACTTTATGAG
AGGTACATGGTGATTATAAAGAACAATTTCTCGGACTTCTCGGAAGTTTTTAACAATTGT
CCTGATTTTGGGTTGATAGACATGGACAAAGGACATTATTTGGTTAGACCCAAAACGCAT
ATAGGACCACATTTGTATGCTTTTGATGGGGTTGAGATGATAGATATCTCTGGAGCCGTG
GACGATTCTAAACGGGATGTTCCTGGGATTAAGGTTAATTGGTTGAAACCGGGGTTCTTT
CATGGTATTATGGCTATAAACAAAAACACTATATTGGTTAATGGTCTGATTATAGGAGAT
AGGTTATCTAGGTATTCTCCTTTGGATCTTAGAGGTGACTTGGATGTTGAGTTTATAGAA
GGGCCTCCAGGCTGTGGTAAAACGGAGTTTCTTATATCTAATCATGAGTTTTCACTTACT
GATACTCGGCATATTATTCTGACTGCCTCTAGAGAGGCTTCTGGTGACCTTCGGCGTCGT
GTTACTAACAGATTTCTTACGGACAAAGATATGAAGAAAAGGCGCAATAAAGAGATAATG
AGTATGCGTTATAGGACCATTGATTCCTTCTTGATGCACTTTAAGGAGGGTGTGTATAAA
GTTGACACTCTTTGGATTGATGAGGGTCTTATGAAACATTATGGCGATATAATGTGGTGT
GCTAAATTATCAGGGGCGTCGAAAGTTAAGATTATAGGGGACAGGTCTCAAATTCCTTTT
ATTAATAGAGTTGCGGGTATAGAGTTGAAATATCATTTGGTGCCTTCGTCAGGTATCAAT
TTACGAGTTTTGAATGTGTCTTATAGGTGTCCTTTAGATGTAGTCGCGTTACTAAATTCA
TATGGTAATTACGGGGATAAAGTATATGGTACTTCCAAGGTGCGGAGGAGTATAACGTTG
GAAGAGATTTCTAGTTTGGCGGCTGCGAGGAACGCCATAGTTAAAGCAGATAGGGTGCTA
GTTTTTACTCAGTCTGAGAAAGCAGAGGTTGCTACACTTACTGGTAGAGTGTCAACTATA
AATGAGTATCAGGGTTCGCAAGCCGAGAATGTTGTATGTATTCGTCTGAATAAGAAGAAA
AATGAGGTGTATGATTCCATGAATCATATAATAGTGGCTATAAGTAGACATACTAAGTGT
TTCACTTATTTTACCGTTTCCAGGGACAGAATGTATAACATATTGTCTACTCAGATTTCT
AATGCTACTGTTGATGACTGTAAGTATGTTCATGATAAGATGCGGGGTGGATATATAGAT
GAGTCACCTGAAAATGAGTGTATTAGGAAAGTTCCGATATCGGGTTATGAGTTGGGGTTG
GATATATACAATTCGGATAGTGCTCAGGGTGCCTTTTATAATTTACGGTATGTACCACCC
ACAACTTTAGATCAGATGAAATTTGATAGTGGGATTCGAAATTTTGTTCAACGTCATGCC
GAGTATGGGGTGGTACCGGCCACGTATATAAAGACAGGCATCGATATTCCCATTGTGGGT
AGGACAGATCACGGTGTTGAAAAGGGGTGTGGTTCTGTAGTCCCGCGGGTGCATGTATTG
CAACATTTTCATGATGAGTTGATTGAGGGTATGTCTATTATTCCCACGGCTTATGATGGA
AAGATTTTTGAAATTGATGAGTTGCATTTACCTAAAGCTGACAATATTAGGTTTTCTACT
GTCTTTCATACACGGATGGGAAAATACGATACTTTGACTCCAGTAGTGCGTACTTCGTGC
CCAGAACCAGTTGTTAGTACATTTAGGCAAACGGTTAAAGGGTTTTTTGACAGGAACGGG
GCTGTACCTGAGTTGCAGGGGATGGTTGATGATTATAGAATTGCTGAAGACACATTGGTT
AGTTTTATAGAGACATACGTTGGTAATTTGGAGGTATTTCGGACTTATGAAGAAAATCCT
GTTTTTATTAATGTACCACAATTGGAGATGTGGTTGAAAGGGCAATCTAGTGCTGTTTAT

GGGGTTATGCAGGATGAAACTTATTGGAGTGTTTTAGATCGTAAGATTTCTGCATACGAT
TTTATATTAAAAAGAGTTCCTAAACCTAAACTGGATTCAACCGCAGTGGCAAAATACCCA
TCACCGCAAACCATTGCTCATTTGAGTAAGGATTTTAATGCTGTCTTTTGTCCTATGCTT
AAGGAGTTGCGGCGACGATTGCTTATGGTTTTGGCTGATAGATTTCTTATGTATTCTGAT
GTATCCCCTGAGGAATTTGAAGATTTGCTTACCGTCAGGTTTCCAATAGGGAGAGTGGGT
ACATATTCCCATGTCGTTGAGATTGATATGAGTAAGTATGATAAGAGTCAGAATAGGACG
GCTTTAATTTTTGAAATGTTATTATATAGACGGTTGGGCATGCCAGATTTTTGGTTACAT
ATCTGGATGCGATTGCACGTGTATACAACGTTGATTGATCATCAAAATCAATTTGCAGCT
GATGTTGTTTTCCAGCGGAAGTCTGGGGATGCCGCTACCTTTTTTGGTAATACGGTGTAT
CTAATGGCCATGATGTCTAAGGTTGTAGATTTAACTGATTCGTATGGTCTTTTTTCAGGA
GATGATAGTTTATTATTCAATAGATCTGCTGATGTATGTCGTGGTATTCCTGAAAAACTT
GCATGTAATTATAATATGGAGGCTAAGGTTTTAACATACAAATCATTGTATTTTTGCTCT
AAGTTTCTTATTCCCGGTGTCAATGGCAGGTGGTATTTTATACCAGATCCGGTTAAATTA
TTAGTTAAGTTGGGCAGGAAGGATTTAAAAAATTTTTCTCACGTTGAACAGTACAGAGTG
TCTTATGTTGATTTGGTTGATCTTTACACAGATCCTTTTGTTTACCCTGCTTTGAGTCAA
TCAGTGCGCGACCGATATCGAGAATCCCGCACGGAAGTGACAGACTTCTATTATATGTTT
GCTATGCTACAGGCATATGTTAGGGATGCTGATAGTTTTCGACAGTTATATTATGTTCTC
CCGCATCATGTTATTGATGAGGTATCCGGTGCGAAATTAAACGATATATAAATGGTTATA
TTAATGGGAAAAGATTTTCATTTTGTTGTATTGTTGGTTTTCTCAGTCATGGCTACCTTG
GGAACTCCGCAGCTTGTTGCACGTCGTGCTGTTAATGATGGTGGTCTGCCATCAGACACT
GCGAACTACAGAGGGTATGACTCTCTGGGTAGACCGGCTGTTAGGTCTGGGCAGACGGTT
GGTATAACATTTCCGGATGCTGTCATGGCATCGTATTCTAATTTGGTTTATCATCCGGTG
GCACTGGTTTGTCTTTTCTTTGGTAGTTTGATAGCATTGTATGAGTATGGAAACGCTTAT
GGACCGTTGGAATTGGCTCTTACTAATATTAATAGTTGGGATCCTGGAAATTTTGGTTTT
GTTAAAACAATGTTGTTAAATTTTGTTCGATTGGCAGTGGCAAACAAAATGTTCTTTAGT
AAAACGTTGATGTTATTTTCTATATATTTGGTTAAACCTTCTAATAGAACATTGACGTGG
TTGGCGGTTATGATGATTTATTTCGTAGTCGTTAATATAGATATGATAGAAGCATTTTTG
ATATGTCAGGGTTTATATTTATTGGTTATGTTAAGGAGTCCGGTGCATAAGTTGATTTCT
ATTTTCTTTATTGGTTTGGTTATGATTTTTGATTCAGTTATGTGTAGGTTTGCTGTTTTG
TCTAATTCTACTTAACTAGTTTTGTTTTGTCTTATTGATCCGACCGGTGATCGAATAATT
AGGTTAGTAAATCCGGTTATTTGTTTAAGTTAGGTTTATGTTTTTACTAACTTGTCCATT
ATTATATCTTAAGGTATTTAATTTTCTTTCTTTCTTTCTTAAAAAAAAAAAA
